# Supplementary material for: Cold atmospheric plasma differentially affects cell renewal and differentiation of stem cells and APC-deficient-derived tumor cells in intestinal organoids
Source: Cell Death Discov. 2022 Feb 15;8:66. doi: 10.1038/s41420-022-00835-7 (PMC8847667; doi:10.1038/s41420-022-00835-7)
Supplement: Supplementary file 1 — Supplementary figures text summary [file 41420_2022_835_MOESM1_ESM.docx]

**COLD ATMOSPHERIC PLASMA DIFFERENTIALLY AFFECTS CELL RENEWAL AND DIFFERENTIATION OF STEM CELLS AND APC-DEFICIENT-DERIVED TUMOR CELLS IN INTESTINAL ORGANOIDS**

**Hadefi et al.**

**SUPPLEMENTARY FIGURES Content**

**Figure S1. Impact of the CAP application method on organoid morphology.**

**Figure S2. Impact of the CAP application method on global gene expression of intestinal organoids.**

**Figure S3. Apc deficient-derived organoids exhibit increased resistance to CAP treatment as compared to normal intestinal stem cell-derived organoids.**
