# Supplementary material for: Cold atmospheric plasma differentially affects cell renewal and differentiation of stem cells and APC-deficient-derived tumor cells in intestinal organoids
Source: Cell Death Discov. 2022 Feb 15;8:66. doi: 10.1038/s41420-022-00835-7 (PMC8847667; doi:10.1038/s41420-022-00835-7)

**COLD ATMOSPHERIC PLASMA DIFFERENTIALLY AFFECTS CELL RENEWAL AND DIFFERENTIATION OF STEM CELLS AND APC-DEFICIENT-DERIVED TUMOR CELLS IN INTESTINAL ORGANOIDS**

**Hadefi et al.**

**SUPPLEMENTARY FIGURES Content**

**Figure S1. Impact of the CAP application method on organoid morphology.**

**Figure S2. Impact of the CAP application method on global gene expression of intestinal organoids.**

**Figure S3. Apc deficient-derived organoids exhibit increased resistance to CAP treatment as compared to normal intestinal stem cell-derived organoids.**

**Figure S1. Impact of the CAP application method on organoid morphology. a**. CAP-conditioned media and PAM generated by treatment with 50 W for 60 s at day 1 were applied directly to organoid cultures at day 2 post-replating for 24 hours (until day 3). **b**. Representative pictures of a given field showing growth of organoids at day 2 (before CAP application) and day 3 (endpoint of the experiment). Triangles show individual elements evolving as protruded organoids and spheroids in untreated and CAP-treated cultures, respectively. Right panels: insets of the pictures at day 3. Scale bars: 500 µm. **c**. Quantification of organoid complexity at day 3. An average number of 100 elements was analyzed over time per condition per organoid line (n= 4 organoid lines). Data are represented as means ± sem. Two-way ANOVA: interaction ** P< 0.01 followed by Dunnett's multiple comparisons test: *** P< 0.001, ** P< 0.01, * P< 0.05, ns not significant (all compared to untreated).

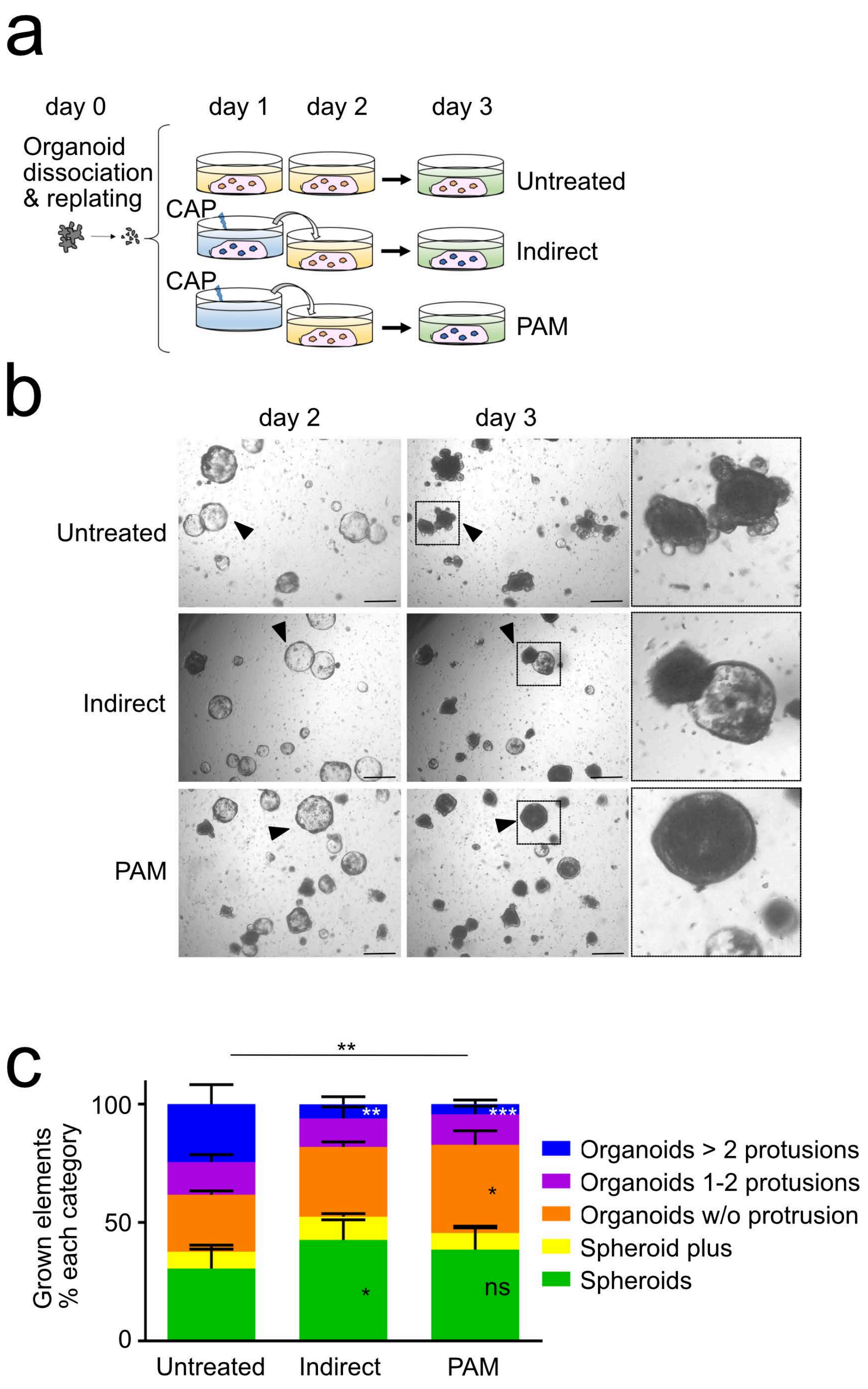

**Figure S2. Impact of the CAP application method on global gene expression of intestinal organoids. a**. Heatmap of the most differentially regulated genes in CAP-treated versus Untreated (Controls) organoids at the early time post-treatment (30 min). **b**. Expression levels of Cys metabolism-, cell signaling-, inflammation- and cytoskeleton organization-associated genes in the various conditions. Data are represented as means ± sd. n = 4 and 3 samples in Controls and CAP-treated conditions, respectively. CP20M: counts per kilobase of transcript per 20 million mapped reads.

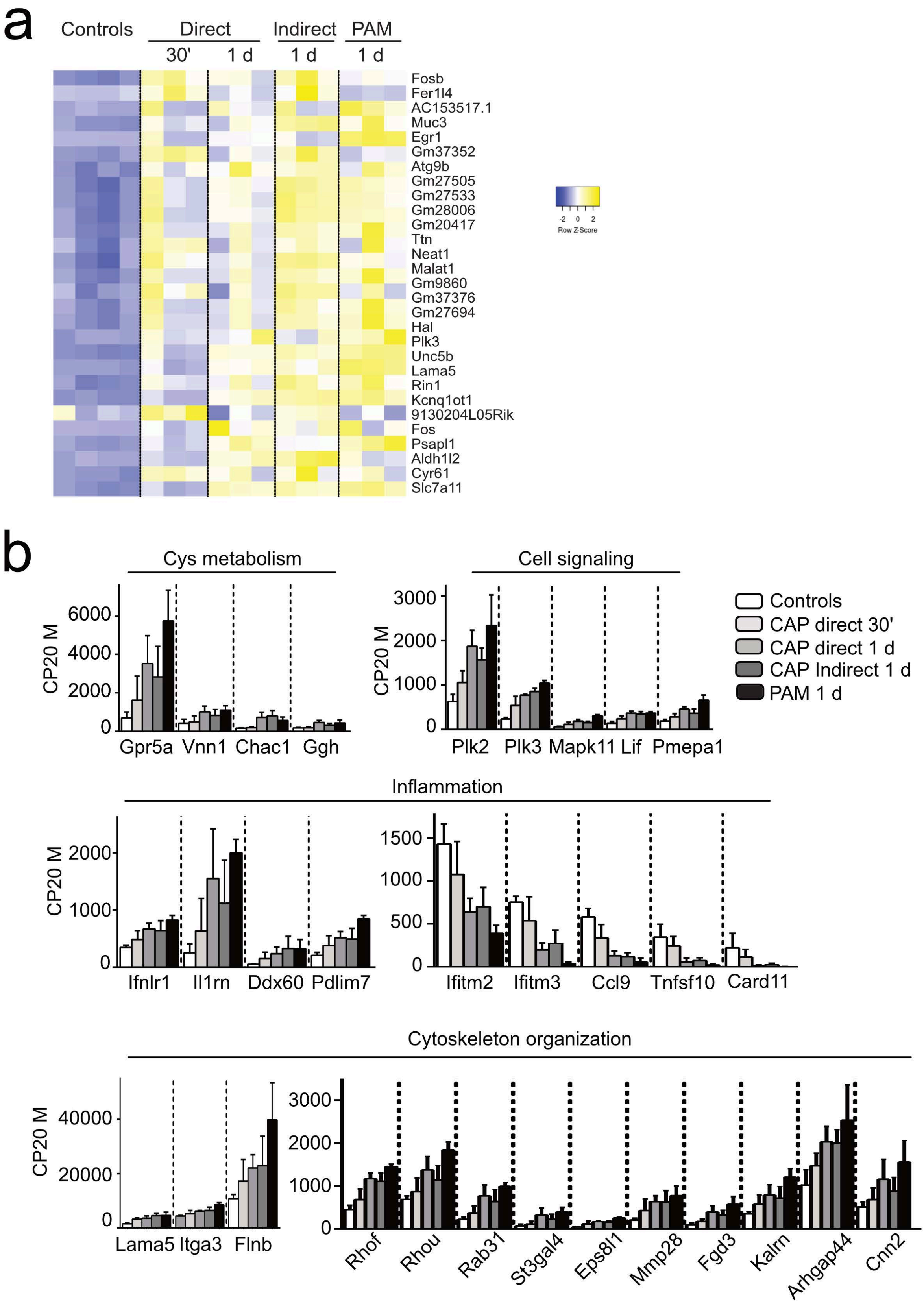

**Figure S3. Apc deficient-derived organoids exhibit increased resistance to CAP treatment as compared to normal intestinal stem cell-derived organoids.**

**a**. Organoid growth of Apc wt and Apc crypts upon initial seeding in culture media containing ENR (EGF, Noggin, Rspondin1) or EN (EGF, Noggin). Representative pictures of a given field showing organoid morphology at day 1 and day 4. Apc Δ organoid efficiently grow in EN conditions as compared to Apc wt organoids. Scale bars: 500 µm. **b**. Measurement of reactive species in culture supernatants of Apc wt and Apc Δ organoids 24 hours after direct CAP treatment or PAM at the indicated doses. DCFDA dye was used to measure ROS levels. A.U. Arbitrary Units. Each symbol corresponds to a given organoid line. Note that measurements of PAM were done in simplicate. Data are represented as means ± sem. One-way Anova test. **** P< 0.0001. **c**. Expression levels of aquaporin-encoding genes. Left panel: Aqp expression in Lgr5+ve ISC. Data were analyzed from the Gene Expression Omnibus GSE135362 dataset; Right panel: Aqp expression in control and CAP-treated organoids (this work). CP20M: counts per kilobase of transcript per 20 million mapped reads (n =2 samples). **d**. Gene expression analysis by qRT-PCR of Aquaporins in Apc wt and Apc Δ organoids. Each symbol corresponds to a given organoid line. Values are normalized to Untreated Apc wt levels. Data are represented as means ± sem. One-way Anova test. **** P< 0.0001; *** P< 0.001. e. Expression levels of ROS scavenger enzymes in Lgr5+ve ISC. Data were analyzed from the Gene Expression Omnibus GSE135362 dataset. CP20M: counts per kilobase of transcript per 20 million mapped reads (n =2 samples).

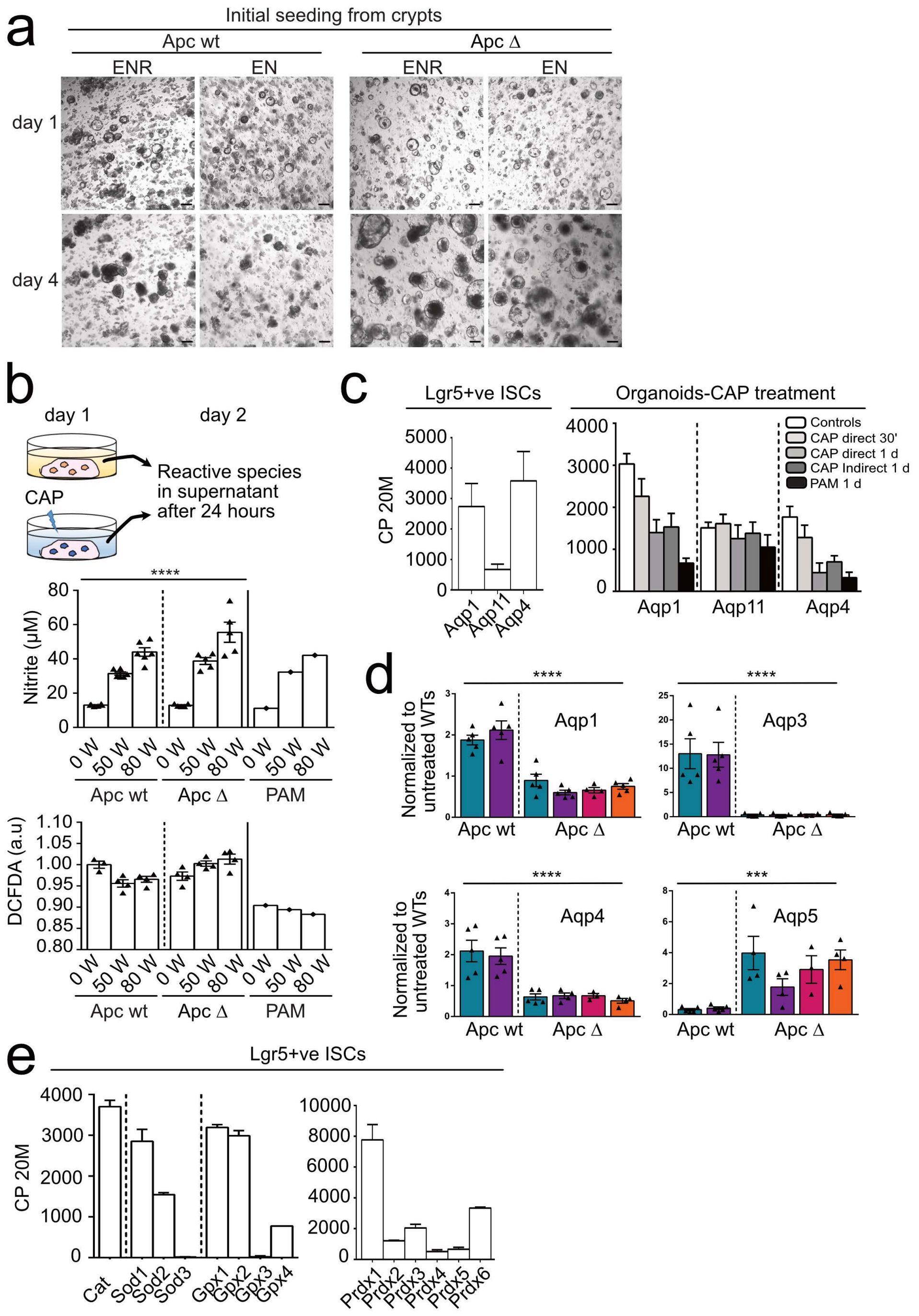

Supplement: Supplementary file 2 — Hadefi et al Supplementary figures-revised ms.pdf [file 41420_2022_835_MOESM2_ESM.pdf]
