# Supplementary material for: Cold atmospheric plasma differentially affects cell renewal and differentiation of stem cells and APC-deficient-derived tumor cells in intestinal organoids
Source: Cell Death Discov. 2022 Feb 15;8:66. doi: 10.1038/s41420-022-00835-7 (PMC8847667; doi:10.1038/s41420-022-00835-7)
Supplement: Supplementary file 3 — Detailed contribution of authorship [file 41420_2022_835_MOESM3_ESM.docx]

**Detailed contribution of authorship**

Section 1 : General terms

AH, ML, OB, MT: study concept and design, acquisition of data, analysis and interpretation of data, statistical analysis, drafting of the ms

AL, FL generated data

JD, AN, AD, FR: study concept and design, study supervision, critical revision of the ms, obtained funding

MIG: study concept and design, acquisition of data, analysis and interpretation of data, statistical analysis, drafting of the ms, study supervision, obtained funding

Section 2 : Figure contribution

***Figure 1***

MT, OB generated the data for panels c,d and prepared panel a

ML generated the data for panels c/d

MIG prepared panel b and assembled the figure

***Figure 2***

ML generated the data for panels b, c, d, e

MT, OB generated the data for panels b, c, d

AH prepared panels d, e

AL, FL generated the data for panels d

MIG prepared panel a, generated the data of panel c and assembled the figure

***Figure 3***

AH prepared the panels a, b, d

ML generated the data for panels c and d

MIG prepared panels c, d and assembled the figure

***Figure 4***

AH prepared the panel a

ML generated the data for panel b

MIG prepared panel b and assembled the figure

***Figure 5***

AH prepared the panel a and b

ML generated the data for panels c and d

MIG prepared panels c, d and assembled the figure

***Figure 6***

AH, ML, MT, OB generated the data for panels b, c, d

MIG prepared panels a, b, c, d and assembled the figure

***Figure 7***

AH, ML, MT, OB generated the data for panel b

MIG prepared panels a, b and assembled the figure

***Supplementary Figure S1***

ML, MT, OB generated the data for panels b, c

MIG prepared panel a, generated the data of panel c and assembled the figure

***Supplementary Figure S2***

ML, AH, MT, OB generated the data for panels a, b

AH prepared panels a, b

MIG assembled the figure

***Supplementary Figure S3***

ML generated the data for panel a, b, d

MT, OB generated the data for panels b, d

AH prepared panels c, e

MIG prepared panel b, d and assembled the figure
