# Supplementary material for: Cold atmospheric plasma differentially affects cell renewal and differentiation of stem cells and APC-deficient-derived tumor cells in intestinal organoids
Source: Cell Death Discov. 2022 Feb 15;8:66. doi: 10.1038/s41420-022-00835-7 (PMC8847667; doi:10.1038/s41420-022-00835-7)
Supplement: Supplementary file 4 — nature-manuscript-checklist-research file filled [file 41420_2022_835_MOESM4_ESM.pdf]

Nature manuscript checklist

Manuscript number. CDDISCOVERY-21-2896R

Author name . . Hadefi et al

Article type . . Research article

The following checklist is intended to aid the publication of your manuscript. Please read through the list carefully and amend your manuscript as appropriate. To avoid delays in the production process, please complete and return this sheet with the final version of your manuscript.

TEXT

Nature will edit text to conform to house style and for clarity and accessibility. Please check the following points, if applicable, when preparing the final version of your manuscript.

- ☒ Title no longer than 75 characters (including spaces)
- ☒ Summary paragraph referenced and ideally no more than 200 words
- ☒ Online-only Methods provided as part of the main text file
- ☒ References as agreed limit
- ☒ Abbreviations and symbols defined on first use
- ☒ Italic font used for variables, genes and non-English languages; bold font for vectors
- ☒ Equations and special characters in Word in 'normal text' or Symbol font (Word Equation Editor avoided where possible)
- ☒ Brief title provided for each figure, table and box
- ☒ Figure legends no more than 300 words each
- ☒ Legends for Extended Data Figures included in the main text file after the online-only Methods
- ☒ Author contribution statement included (a statement of responsibility that specifies the contribution of every author; see https://www.nature.com/authors/editorial_policies/authorship.html)
- ☒ Declaration and listing of any financial or non-financial competing interests included in manuscript (https://www.nature.com/authors/editorial_policies/competing.html)
- ☒ Database accession numbers included for sequences determined
- ☒ Structure data in *Nature* standard tables

FOR TEX USERS

Word or rtf formats are preferred. Such formats enable on-screen subediting and the preparation of a 'pre-proof', when the editing can be checked before formal page proofs are prepared. If you have prepared your paper using TeX/LaTeX, please convert to PDF format and upload the PDF file in addition to the single .tex file at submission. We will convert the .tex file to Word and require the PDF for cross-reference purposes in case anything is unclear.

All textual material of the paper (including references, tables, figure captions, online methods, etc.) should be in electronic form, as a single .tex file.

Contact our subeditors at authors@nature.com for further help and advice on preparing text for publication.

ARTWORK — PRINT-ONLY FIGURES

Nature will edit figures to conform to house style and for clear reproduction in print. Standard figure sizes are 89 mm wide (single column) and 183 mm wide (double column); figures can also be a column-and-a-half width where necessary (120–136 mm). Please check the following points when preparing your figures.

Electronic figure formats

- ☒ Preferred file formats for line (or vector) artwork and graphs are unflattened EPS, postscript, PDF and Illustrator (AI) files
- ☒ Preferred file formats for photographic images are TIFF and Photoshop (minimum resolution 300 dpi). If you have created images with separate components on different layers, please send us the Photoshop file (.psd) with the layers intact
- ☒ For detailed guidelines and a full list of acceptable electronic formats, see https://www.nature.com/documents/nature-final-artwork.pdf

When preparing and sending figures

- ☒ Text in an editable format and in sans serif font (Helvetica, Arial)
- ☒ Text and lines clearly visible when figure is sized for page: type size 6–8 point; lines preferably 0.5 point (0.18 mm)
- ☒ Colour artwork supplied in RGB (recommended) or CMYK formats
- ☒ Suggestions for cover artwork submitted, if desired
- ☒ Licence/permission obtained for any republished/redrawn illustrations

Email our art editors at **art@nature.com** for further help and advice.

ARTWORK — EXTENDED DATA DISPLAY ITEMS

- ☒ *Nature* does not edit these figures. Please ensure that you have followed our guidelines as closely as possible before submission (see https://www.nature.com/documents/nature-extended-data.pdf). All Extended Data files supplied as 300 ppi images, saved as **EPS, JPEG or TIFF file formats only**, with colour images in RGB (recommended) or CMYK formats, and with each display item not more than 10 MB.

SUPPLEMENTARY INFORMATION

Nature does not edit online-only material. Please check the following points when preparing Supplementary Information:

- ☒ Discrete items of the Supplementary Information (for example, videos) referred to at an appropriate point in the printed text
- ☒ Separate text file ('SI Guide') provided with a brief description (file format and content, video legends) of each item
- ☒ We recommend that all Supplementary Information is supplied as a single PDF file, where possible.
- ☒ File size within the permitted limits for Supplementary Information.

See https://www.nature.com/nature/for-authors/supp-info for advice on preparing Supplementary Information.

E-mail authors@nature.com if you have additional queries.
